# Deep learning-based breast cancer detection with customized ensemble attention

**DOI:** 10.1038/s41598-026-50563-6

**Published:** 2026-04-29

**Authors:** Divyakant T. Meva, Kalpesh Popat, Hirenkumar Kukadiya, Nikisha Jariwala

**Affiliations:** 1 Faculty of Computer Applications, Marwadi University, Rajkot, Gujarat India; 2 Institute of Computer Science and Applications, Gandhinagar University, Gandhinagar, Gujarat India; 3 Smt. Tanuben & Dr. Manubhai Trivedi College of Information Science, Veer Narmad South Gujarat University, Surat, Gujarat India

**Keywords:** Breast cancer, Deep learning, Ensemble learning, Customized attention mechanisms, Multi-Scale Channel Attention, Spatial-Morphological Attention, Hierarchical Dual Attention, Histopathology, Mammography, BreakHis; BACH, CBIS-DDSM, Cancer, Computational biology and bioinformatics, Mathematics and computing

## Abstract

Breast cancer remains one of the leading malignancies globally, and accurate diagnostic decisions at the early stages of the disease can significantly improve patient prognosis. This paper introduces a new ensemble deep learning framework, combining three specific attention mechanisms tailored to the breast pathology domain, namely, Multi-Scale Channel Attention (MSCA), Spatial-Morphological Attention (SMA), and Hierarchical Dual Attention (HDA). Unlike generic SENet and CBAM mechanisms, these modules are specifically designed for breast pathology: MSCA captures nuclear-level channel statistics across multiple pooling scales, SMA applies learnable morphological gradient operators to tissue boundary regions, and HDA employs depth-dependent gating to dynamically balance spatial and semantic attention across network stages. While domain-adaptive attention has been explored in medical imaging, the specific combination of multi-scale nuclear statistics, learnable morphological gradients, and depth-adaptive gating tailored jointly to histopathological and mammographic imaging represents a distinct and novel architectural contribution. The framework incorporates three complementary CNN backbones (ResNet-50, DenseNet-121, and EfficientNet-B3) augmented with the proposed attention modules, and intelligently fuses their predictions using a confidence- and performance-based weighted ensemble strategy. Comprehensive experiments were conducted on three publicly available benchmark datasets: the BreakHis histopathology dataset (with 1,995 different images at 40× magnification), the BACH challenge dataset (with 400 WSIs producing 9,600 patches), and the CBIS-DDSM mammography dataset (with 2,620 cases). The proposed framework yields statistically significant (*p* < 0.05; paired t-test across five folds) improvements upon individual backbone constituents and surpasses attention-augmented baselines SENet and CBAM by up to 1.8 pp on BreakHis (95.6 ± 0.5% acc), BACH (91.3 ± 0.8% acc), and CBIS-DDSM (93.7 ± 1.0% acc) Ablation studies confirmed the contribution of each attention module (MSCA + 1.6%, SMA + 1.2%, HDA + 1.0%) individually, and a clinical reader study revealed that AI assistance significantly improved the accuracy of pathologists on more challenging cases (accuracy increasing from 85.4 to 90.2%; *p* = 0.012). Attention visualizations exhibited clinically meaningful attention overlap with pathologist-annotated ROIs (mean IoU = 0.76) compared to generic CBAM-based attention (IoU = 0.59).

## Introduction

Breast cancer is the most common malignancy among women worldwide. The global cancer observatory has reported 2.3 million new cases and 685,000 deaths in 2020, alone, with breast cancer accounting for approximately 12.5% of all new cancer cases worldwide in 2024. The five-year survival rate is over 90% for the localized disease and approximately 28% for the distant metastatic disease^1^. Molecular studies have further revealed that metabolic reprogramming—including autophagy-mediated degradation of key metabolic enzymes—plays a critical role in breast cancer progression, underscoring the biological complexity that computational diagnostic systems must ultimately account for^2^. Therefore, the role of diagnosis in this malignancy is highly imperative^3–5^.

Standard diagnostic modalities include mammography, ultrasonography, magnetic resonance imaging (MRI), and histopathological analysis of biopsied tissues^6^. All the modalities have limitations. Mammographic sensitivity is 75–92% depending on breast density and reader experience^7^, while inter-observer agreement among pathologists for borderline lesions is 75–85%^8^. Finally, a global shortage in trained radiologists and pathologists—most notably in low- and middle-income countries—further worsens delays and errors in breast cancer diagnostics^9^.

Deep learning, in particular, Convolutional Neural Networks (CNNs), managed to outperform medical image analysis by enabling automated feature extraction and pattern recognition at the expert level of performance on well-defined benchmark tasks^10,11^. CNNs have shown strong performance on visual recognition benchmark tasks^12^ and have had wide applications in breast cancer classification^13,14^. However, generic CNN architectures are constrained by information loss during downsampling, susceptibility to spatial pooling artifacts, and insufficient focus on diagnostically relevant regions^15^.

Attention mechanisms address these limitations by enabling models to selectively weight feature channels and spatial regions, given diagnostic relevance^16^. Squeeze-and-Excitation Networks (SENet)^17^ and the Convolutional Block Attention Module (CBAM)^18^ are seminal works in channel and spatial attention for boosting classification outcomes. However, these mechanisms are tailored to natural image tasks and are not optimized to capture domain-specific morphological features pertinent to breast pathology, such as nuclear pleomorphism, mitotic figures, and microcalcification patterns^3^. Specifically, CBAM assumes fixed sequential channel-then-spatial attention without multi-scale channel aggregation or morphological boundary awareness, while SENet entails single-channel recalibration from a single pooling and has no spatial or depth-adaptive capacity. The proposed MSCA, SMA, and HDA modules cater for such deficiencies in multi-scale pooling, learnable morphological gradients, and depth-dependent gating, respectively^19^^[,20^.

Ensemble learning, which combines predictions from multiple models to reduce variance and improve generalization, has shown consistent gains over single-model approaches in breast cancer diagnosis^21,22^. Nevertheless, the synergistic combination of domain-specific attention mechanisms within a heterogeneous ensemble architecture has received limited investigation. Furthermore, the ImageNet-to-pathology domain shift poses a known challenge for transfer learning: while low-level texture features transfer reasonably well, features encoding natural image semantics may not map to histopathological patterns such as nuclear pleomorphism or microcalcification distributions. The proposed framework explicitly addresses this through domain-adaptive attention design and a two-stage fine-tuning procedure that progressively unfreezes layers to reconcile pretrained representations with breast-pathology feature requirements. Most existing ensemble strategies use homogeneous base architectures or employ simplistic aggregation methods that do not leverage the complementary nature of diverse attention mechanisms^23^^[,24^.

To address these research gaps, this paper proposes a novel ensemble deep learning framework integrating three breast-pathology-specific attention modules within architecturally diverse CNN backbones, combined through an intelligent confidence- and performance-based ensemble strategy. The specific contributions are as follows:

- Proposed three novel attention modules (MSCA, SMA, HDA) specific to the morphological characteristics of breast cancer imaging offer advantages over standard mechanisms (CBAM, SENet), addressing multi-scale channel aggregation, structural boundary detection, and depth-adaptive attention fusion.
- A heterogeneous ensemble architecture with ResNet-50, DenseNet-121, and EfficientNet-B3, each integrated with the proposed attention modules, ensuring diversity of the architectures and complementary feature spaces.
- An ensemble aggregation strategy on a confidence- and performance-weighted intelligence that dynamically assigns weights to the models based on the entropy of the prediction and weights based on the best performing class weights with respect to the validation set F1 scores.
- Extensive evaluation, including ablation studies, cross-dataset generalization experiments, computational efficiency analysis, and a clinical reader study over three benchmarks datasets (BreakHis, BACH, CBIS-DDSM).

The remainder of this paper is as follows; Sect. 2 reviews related literature and their research gaps. Section 3 describes the proposed methodology: preprocessing, attention modules, backbone architectures, ensemble strategy, and training procedure. Section 4 mentions the experimental setup, datasets, and baselines. Section 5 provides a comprehensive presentation and analysis of the results. Section 6 presents a discussion on findings, limitations, and future directions. Section 7 presents the conclusion.

## Literature review

### Breast cancer imaging modalities and diagnostic challenges

Mammography still bears the largest deployed screening modality for breast cancer. Digital mammography uses additional sensitivity modules for dense breast tissue and has been augmented by computer-aided detection modules^6,7^; tissue superposition reduces sensitivity in up to 75% of extremely dense breasts^7^. The diagnostic gold standard remains the histopathological evaluation of hematoxylin and eosin (H&E) tissue samples, which provides for the assessment of cellular morphology, nuclear grade, and tumor architecture^8,25^. Whole-slide imaging (WSI) has enabled in the digitization of pathology workflows and remote consultations, but WSI files may reach gigapixel resolutions, which presents significant computational challenges for automated pipelines^26^.

### Deep learning in breast cancer diagnosis

Deep learning has substantially advanced breast cancer image analysis since early demonstrations that CNNs could match radiologist performance on mammographic lesion detection^13^. More recently, dual-branch CNN architectures have demonstrated competitive performance on breast ultrasound image detection, further broadening the scope of deep learning to multi-modal breast imaging^27^. The CAMELYON16 challenge showed that deep learning systems could reach pathologist-level accuracy in identifying lymph node metastases^28^. For invasive ductal carcinoma detection in WSIs, Cruz-Roa et al.^15^ achieved 75% patch-level sensitivity. Araújo et al.^14^ demonstrated that well-regularized deep CNNs outperform shallow networks for breast histopathology classification. More recently, transformer-based architectures applied to computational pathology have achieved high accuracy on glioma classification^28,]^, and hybrid CNN-transformer ensemble models have enhanced multi-class cancer classification^29,30^.

### Attention mechanisms in deep learning and medical imaging

Attention mechanisms originated in neural machine translation^16^ and were formalized in the Transformer architecture^31^. In CNNs, SENet^17^ introduced computationally efficient channel-wise feature recalibration. Non-local neural networks^32^ extended this to long-range spatial dependencies. CBAM^18^ combined sequential channel and spatial attention with steady improvements on image classification benchmarks. In medical imaging, attention gates^4^ enabled end-to-end trainable segmentation without external supervision. Dual-path attention networks applied to breast histopathology demonstrated improved classification and interpretable attention maps^3^. Despite these advances, most medical imaging attention architectures employ generic mechanisms without domain-specific design features, such as nuclear morphology cues relevant to histopathology or microcalcification spatial distributions relevant to mammography^3,5^.

### Ensemble learning for medical diagnosis

Ensemble methods reduce prediction error by aggregating diverse learners^21^. Alom et al.^33^ reported strong performance on the DDSM database using weighted averaging of Inception variants.

Yan et al.^34^ developed a multi-view ensemble exploiting complementary craniocaudal and mediolateral oblique projections, which increased accuracy by 4–6% compared to single-view baselines. Hameed et al.^35^ have explored heterogeneous deep learning ensembles in a more extensive manner with consistent improvements with various breast cancer datasets. Kim et al.^36^ proposed meta-learner-based adaptive weighting with performance gain over fixed-weight ensembles. However, for breast cancer diagnosis, most existing ensemble designs use homogeneous base architectures or lack explicit complementary attention across the models’ components, thus underexploiting diverse feature spaces.

### Federated and explainable AI in breast cancer

The AI framework for breast cancer is no longer limited to classifying data from a single institution. Hybrid federated learning with vision transformers was found promising for breast cancer risk prediction without compromising patient data privacy^37^. Transfer learning-based explainable ensemble methods have been validated for OCT-based disease detection, demonstrating the real-life utility of interpretability-driven ensemble design^38^. These developments point toward a new generation of breast cancer AI systems that must simultaneously address performance, generalizability, interpretability, and data privacy.

### Summary of prior work and research gaps

Table 1 summarizes representative prior studies on deep learning for breast cancer detection, highlighting datasets, methods, key results, and limitations relative to the proposed work.

**Table 1:** Summary of representative prior studies on deep learning-based breast cancer detection and classification.

Year	Dataset	Method	Results	Key Limitation	Gap Addressed by This Work
2016	BreakHis	Spanhol et al.: Ensemble CNNs	Acc: 90.8%	Homogeneous ensemble; no attention	Heterogeneous ensemble + customized attention
2017	Histopath.	Araújo et al.: Multi-scale CNN	F1: 83.2%	Single architecture; no ensemble	Multi-backbone ensemble with complementary attention
2017	CAMELYON16	Bejnordi et al.: DL ensemble	AUC: 0.994	Lymph node only; not generalizable to subtypes	Multi-dataset evaluation across histology and mammography
2020	BreakHis	Zhang et al.: Dual-path attention	Acc: 95.2%	Generic attention; not breast-specific	MSCA/SMA/HDA tailored to breast pathology morphology
2021	BreakHis/BACH	Li et al.: Pyramid attention	Acc: 95.8%	Single backbone; limited ensemble diversity	Three complementary backbones with architectural diversity
2021	DDSM	Kim et al.: Meta-learner ensemble	Acc: 96.3%	No customized attention; limited interpretability	Domain-specific attention + clinical attention visualization
2022	BreakHis	Hameed et al.: Heterogeneous ensemble	Acc: 94.7%	Generic attention; no domain optimization	Breast-pathology-specific attention across all backbones
2024	FFDM	ETECADx: Transformer ensemble	AUC: 0.932	High compute cost; limited histopath. evaluation	Efficient CNN ensemble with cross-dataset generalization
Proposed	BreakHis, BACH, CBIS-DDSM	Ensemble + MSCA/SMA/HDA	Acc: 95.6%	Benchmark-dataset validation; compute-intensive	Addresses gaps (i), (ii), (iii) identified in Sect. 2.6

Analysis of the prior literature reveals three principal research gaps. First, most attention mechanisms are designed for natural image tasks and are not specialized for breast pathology morphological characteristics, including nuclear pleomorphism and microcalcification patterns. Second, existing ensemble methods generally do not exploit complementary attention types across architecturally diverse models. Third, interpretability validation against clinical pathologist annotations remains limited. The proposed framework directly addresses all three gaps through domain-specific attention design, heterogeneous ensemble architecture, and clinical reader validation.

## Methodology

### Overview of proposed framework

The proposed deep learning framework comprises four integrated components: (1) a preprocessing and data augmentation pipeline adapted for breast pathology imaging; (2) three customized attention modules designed for the morphological characteristics of breast tissue; (3) three CNN backbone architectures each augmented with the proposed attention modules at strategically motivated positions; and (4) an intelligent ensemble aggregation strategy combining confidence-based and performance-based weighting.

Three parallel processing streams accept input images through ResNet-50, DenseNet-121, and EfficientNet-B3 as backbone models. MSCA, SMA, and HDA modules are incorporated at stage-specific positions within each backbone. The ensemble strategy aggregates outputs from all three augmented models to produce final diagnostic predictions. A workflow summary is provided in the subsections below, with full architectural detail given in Appendix A.

### Preprocessing and data augmentation

First, all input images are resized to 224 × 224 pixels and normalized using ImageNet mean (µ = [0.485, 0.456, 0.406]) and standard deviation (σ = [0.229, 0.224, 0.225]). For histopathology images, Otsu thresholding extracts tissue regions from background, and Macenko color normalization^39^ is performed to normalize staining differences across slides with a dataset-specific reference image. For mammography images, local contrast is enhanced without raising noise levels using contrast-limited adaptive histogram equalization (CLAHE) with a clip limit of 2.0 and a tile grid size of 8 × 8. To assess the individual contribution of preprocessing steps, an ablation was conducted on BreakHis: removing Macenko normalization reduced accuracy by 1.1% (94.5% vs. 95.6%), removing CLAHE on mammography reduced CBIS-DDSM accuracy by 0.9%, and removing tissue-region extraction reduced accuracy by 0.6%, confirming that each preprocessing step provides a measurable, additive benefit to downstream classification performance.

Stochastic augmentations used during training include random flips horizontally and vertically (*p* = 0.5 each), a random rotation (± 15°, *p* = 0.7), a random affine transformation (shear range ± 0.2, *p* = 0.5), a color jitter (brightness ± 0.2, contrast ± 0.2, saturation ± 0.1, *p* = 0.8), a random Gaussian blur (kernel 3 × 3, σ = 0.1–2.0, *p* = 0.3), and a random erasing (*p* = 0.2, scale range 0.02–0.2, ratio range 0.3–3.3). The transformations are based on realistic image acquisition inconsistencies encountered in clinical practice such as staining variability, tissue orientation in the image, and partial occlusion artifact.

### Customized attention mechanisms

#### Multi-scale channel attention (MSCA)

The MSCA module tackles the multi-scale nature of diagnostically pertinent characteristics in breast histopathology, which ranges from malignancy-indicating aberrations at the nuclear level to abnormalities in the tissue architecture. In contrast to SENet’s single global average pooling operation, MSCA captures channel statistics of three spatial scales at the same time, allowing for the representation of cellular texture details and tissue architecture simultaneously. Given input feature maps X ∈ ℝ^{C×H×W}, three scale-dependent channel-wise statistics are calculated:

$$z_k^s = (1/{H_s}{W_s})\sum\nolimits_{i,j} {{X_c}[i,j],s\varepsilon 1,2,3}$$

where H_s and W_s are the spatial dimensions of pooling kernels of size 1 × 1, 3 × 3, and 5 × 5 respectively (padded to maintain C-dimensional output). The concatenated multi-scale statistics are processed through a bottleneck excitation network:

$$s = \sigma ({W_2}{\text{ }} \cdot \delta ({W_1}{\text{ }} \cdot Concat({z^1},{z^2},{z^3})))$$

where W_1 ∈ ℝ^{(3 C/r)×C}, W_2 ∈ ℝ^{C×(3 C/r)}, reduction ratio *r* = 16, δ denotes ReLU activation, and σ is the sigmoid function. The recalibrated output is:

$$X{{ }} = {{ }}s \otimes X$$

The multi-scale pooling design captures texture-level statistics (small kernels) and structural-level statistics (large kernels) at the same time to account for cellular and tissue-level diagnostic features, respectively. It is a direct architectural advance over single-scale mechanisms: MSCA incurs an overhead of about 11% more parameters over baseline while still rendering a 1.6% accuracy gain on BreakHis (Table 4).

#### Spatial-morphological attention (SMA)

The SMA module integrates learnable morphological operations to highlight structural features at tissue boundaries and cellular junctions, which are diagnostically critical in breast pathology. Unlike standard spatial attention (e.g., CBAM’s average/max-pooled spatial attention), SMA employs learnable structuring elements that adapt to pathological morphological characteristics through backpropagation, rather than relying on fixed hand-crafted kernels. For input feature map X, morphological dilation and erosion are approximated as:

$$X\_\left\{ {dilate} \right\}{\text{ }} = {\text{ }}max - pool\left( {Conv\_\left\{ {3 \times 3} \right\}\left( X \right),{\text{ }}kernel = 3} \right)$$

$$X\_\left\{ {erode} \right\}{\text{ }} = {\text{ }}min - pool\left( {Conv\_\left\{ {3 \times 3} \right\}\left( X \right),{\text{ }}kernel = 3} \right)$$

The morphological gradient emphasizes structural boundaries:

$$X\_\left\{ {morph} \right\}{\text{ }} = {\text{ }}X\_\left\{ {dilate} \right\}{\text{ }} - {\text{ }}X\_\left\{ {erode} \right\}$$

This gradient map is concatenated with standard pooled spatial statistics to generate the spatial attention map:

$$s\_\left\{ {spatial} \right\}{\text{ }} = {\text{ }}\sigma \left( {Conv\_\left\{ {7 \times 7} \right\}\left( {Concat\left( {AvgPool\left( X \right),{\text{ }}MaxPool\left( X \right),{\text{ }}X\_\{ morph\} } \right)} \right)} \right)$$

$$X{{ }} = {{ }}s\_\left\{ {spatial} \right\} \otimes X$$

The learnable morphological operations adapt structuring elements end-to-end to pathology-specific structural characteristics. Compared to classical morphology with fixed kernels, this allows the module to automatically detect the types of boundary features—glandular margins, nuclear envelopes, fibrous stromal boundaries—most discriminative for the training task, without requiring manual kernel design. SMA contributes 1.2% accuracy improvement over the no-attention baseline on BreakHis (Table 4).

#### Hierarchical dual attention (HDA)

Motivated by the hierarchical nature of pathological feature representations, the HDA module combines channel and spatial attention in a depth-adaptive manner. Not only does spatial localization of tissue structures matter most in low-level texture and edge information captured by shallow network layers, but semantic diagnostic concepts encoded in deeper layers (e.g., grading features of carcinomas) also require channel-wise selections as a more important factor. This depth-dependent strategy distinguishes HDA from fixed-ratio attention combinations in CBAM and similar mechanisms. For input features X at network depth d, channel attention Achannel and spatial attention Aspatial are calculated as follows:

$$A\_\left\{ {channel} \right\}{{ }} = {{ }}\sigma \left( {FC\_2\left( {ReLU\left( {FC\_1\left( {GAP\left( X \right)} \right)} \right)} \right)} \right)$$

$$A\_\left\{ {spatial} \right\}{\text{ }} = {\text{ }}\sigma \left( {Conv\_\left\{ {7 \times 7} \right\}\left( {Concat\left( {AvgPool\left( X \right),{\text{ }}MaxPool\left( X \right)} \right)} \right)} \right)$$

A depth-dependent gating scalar λ_d ∈ [0,1] determines the relative weighting of each attention type:

$$\lambda \_d{\text{ }} = {\text{ }}\sigma \left( {w\_d{\text{ }}\cdot{\text{ }}d{\text{ }} + {\text{ }}b\_d} \right)$$

w_d and b_d are scalar parameters that are learned end-to-end. As d increases, λ_d naturally shifts towards higher values, increasing channel attention weighting at these deeper layers where channels are more informative semantically. The intuition is visualizable with emphasis laid on spatial localization, with the final channel selection emphasized, with λd ≈ 0.2, at the depth, d = 1 (early stage), and λd ≈ 0.8 at d = 4, which is the late stage. Finally, the combined attention is applied in sequence:

$$\,A{\text{ }} = {\text{ }}\lambda \_d{\text{ }}\cdot{\text{ }}A\_\left\{ {channel} \right\}{\text{ }} + {\text{ }}\left( {1{\text{ }} - {\text{ }}\lambda \_d} \right){\text{ }}\cdot{\text{ }}A\_\left\{ {spatial} \right\}$$

$$X\_\left\{ {channel} \right\}{\text{ }} = {\text{ }}A\_\left\{ {channel} \right\} \otimes X;\;\;X{\text{ }} = {\text{ }}A\_\left\{ {spatial} \right\} \otimes X\_\left\{ {channel} \right\}$$

This calibrated attention strategy offers more flexibility than fixed-ratio blends by allowing the model to automatically point out whether spatial or semantic attention should get more emphasis on the learned information regarding the depth of features. HDA contributes to 1.0% accuracy over the no-attention baseline on BreakHis (Table 4).

### Base CNN architectures

#### ResNet-50 with attention integration

ResNet-50 is selected as the first base architecture for its residual skip connections that mitigate the vanishing gradient problem and preserve both low-level edge features and high-level semantic representations. MSCA modules are inserted after the second and third residual blocks, where multi-scale channel features at intermediate semantic levels are richest. SMA modules are placed after the first and fourth residual blocks to emphasize morphological characteristics at lower and higher semantic levels, respectively. The HDA module precedes global average pooling, providing depth-adaptive attention fusion at the deepest representation level. This staged insertion strategy reflects the progressive semantic abstraction across residual stages and avoids redundancy by assigning each attention type to the layers where its inductive bias is most beneficial.

#### DenseNet-121 with attention integration

DenseNet-121 is chosen for its densely connected form, in which feature maps for all preceding layers are received by every layer in the network. This ensures forward and backward signal propagation and feature reuse, which is helpful for medical imaging tasks with a limited amount of annotated data. The MSCA modules are integrated into Dense Blocks 2 and 3, leveraging their naturally accumulated multi-scale concatenated features. At the entry and exit points of the dense connectivity network, morphological emphasis is offered by locating SMA modules in Dense Blocks 1 and 4. In this regard, DenseNet-121 supplements ResNet-50 based on its fundamentally different feature aggregation scheme of encoding cross-layer feature dependencies that additive skip connections could not capture.

#### EfficientNet-B3 with attention integration

EfficientNet-B3 is selected for its compound scaling approach that jointly optimizes network depth, width, and input resolution for high performance with a compact parameter count. Its Mobile Inverted Bottleneck Convolution (MBConv) blocks already include Squeeze-and-Excitation channel attention. The proposed framework replaces the SE blocks in Stages 3 and 5 with MSCA modules to deliver richer multi-scale channel attention. SMA modules are inserted in Stages 2 and 6 to enhance spatial-morphological feature awareness. The computational efficiency of EfficientNet-B3 ensures that the ensemble’s architectural diversity does not incur excessive computational overhead. The three architectures—with additive skip connections (ResNet-50), concatenative dense connectivity (DenseNet-121), and compound-scaled inverted bottleneck convolutions (EfficientNet-B3)—provide complementary feature hierarchies and less correlated errors, as confirmed by the architecture diversity ablation in Sect. 5.2.3.

### Ensemble aggregation strategy

#### Confidence-weighted ensemble

Each base model i produces a probability distribution p_i = [p_i^1, …, p_i^K] over K diagnostic classes. Prediction entropy H(p_i) serves as an inverse measure of model confidence:

$$H({p_i}) = - \sum\nolimits_k {p_i^klog(p_i^k)}$$

The confidence weight for model i is:

$$w_i^{conf} = exp( - H({p_i}))/\sum\nolimits_j {exp( - H({p_j}))}$$

Models producing low-entropy (high-confidence) predictions receive higher weighting, enabling the ensemble to dynamically favor the most certain model for each input instance. This approach differs from fixed-weight ensembles^36^ in that weights are recomputed per instance, accommodating varying model confidence across different case types.

#### Performance-based weighting

A running estimate of each model’s class-specific F1-score F1_i^k on the validation set is maintained. For a given test prediction, the performance weight considers the predicted class:

$$w_i^{perf} = F1_i^{argmax({p_i})}/\sum\nolimits_j {F1_j^{argmax({p_j})}}$$

This reflects each model’s historical diagnostic reliability for the specific class being predicted, compensating for models that may be highly confident but systematically less accurate for particular diagnostic categories—a limitation not addressed by confidence weighting alone.

#### Combined ensemble prediction

The final ensemble prediction combines confidence and performance weights through a learned trade-off parameter λ ∈ [0,1]:

$${w_i} = \lambda \cdot w_i^{conf} + (1 - \lambda ) \cdot w_i^{perf}$$

$$w_i^{final} = {w_i}/\sum\nolimits_j {{w_j}}$$

$${p_{ensemble}} = \sum\nolimits_i {w_i^{final}} \cdot {p_i};\overset{\lower0.5em\hbox{$\smash{\scriptscriptstyle\frown}$}}{y} = argmax({p_{ensemble}})$$

The parameter λ = 0.6, selected via grid search on the validation set (Appendix B), slightly favors confidence weighting. Ablation experiments (Table 5) show that the combined strategy consistently outperforms confidence-only (+ 0.5%) and performance-only (+ 0.7%). The computational overhead of this aggregation is negligible (1.2 ms is the additional time required to infer a single image). In comparison to stacking and meta-learning ensemble weighting approaches^36^, the proposed approach is computationally lighter, but largely aggregating quality can be achieved by combining confidence based on entropy with performance signals for each class in a novel way.

### Training procedure

#### Transfer learning and fine-tuning

All base models are initialized with ImageNet-1 K pre-trained weights from the torchvision model zoo and thus benefit from transferable low-level visual features. The training is done in two stages. In Stage 1 (10 epochs), backbone weights are frozen while attention modules and classification heads are trained with a learning rate of 1 × 10^{−4}, allowing attention mechanisms to adapt to features of breast pathology without disrupting already learned representations. In Stage 2 (40 epochs), all layers are unfrozen with discriminative learning rate: initial backbone layers have lr = 1 × 10^{−5}, while later layers and attention modules have lr = 1 × 10^{−4} considering the gradual decrease in transferability from typical significance to task-related feature representations.

#### Loss function and class imbalance handling

Focal loss is employed to handle class imbalance and emphasize hard examples:

$${L_{focal}} = - {\alpha _t}{(1 - {p_t})^ \wedge }\gamma log({p_t})$$

Here, p_t stands for the prediction probability for the ground truth class, αt is a class specific balancing weight based on inverse class frequencies (αt = N/(K · n_t) where N is the total number of samples, K is the number of classes and nt is the sample count in the class t), and γ = 2 is the focusing parameter (grid searched over {0, 1, 2, 3}). Explicit class imbalance statistics are as follows: BreakHis contains 625 benign (31.3%) and 1370 malignant (68.7%) images, BACH – 100 per class (balanced), CBIS-DDSM – 1318 benign (50.3%) and 1302 malignant (49.7%) cases. A diversity regularization term encourages base models to make complementary, non-redundant predictions by penalizing agreement in their output distributions. This is theoretically motivated by the bias-variance decomposition of ensemble error: reducing prediction correlation between models decreases the ensemble variance without necessarily increasing individual model bias. The term is defined as:

$${L_{\left\{ {diversity} \right\}}} = - {\Sigma _{\left\{ {i \ne j} \right\}}}{D_{\left\{ {KL} \right\}}}\left( {{p_i}\parallel {p_j}} \right)$$

The total training loss with diversity weight β = 0.1 is:

$${L_{\{ total\} }} = {L_{\{ focal\} }} + \beta \cdot {L_{\{ diversity\} }}$$

The diversity regularization averts ensemble collapse by penalizing highly correlated predictions between models. Its isolated contribution was assessed in an ablation – removing L_{diversity} reduces BreakHis accuracy by 0.4%, and negatively, increases cross-model prediction correlation by 18%, confirming its role in maintaining prediction diversity. During training, the GPU utilization averaged 92% on the RTX 2000 ADA GPU. The modular attention is scalable to larger backbone variants; thus, memory consumption scales linearly with backbone parameter count.

#### Optimization details

Each base model is trained independently using the AdamW optimizer with β_1 = 0.9, β_2 = 0.999, and weight decay = 1 × 10^{−4}. The learning rate follows a cosine annealing schedule with warm restarts every 10 epochs:

$${\eta _t} = {\eta _{\{ \min \} }} + \left( {{\eta _{\{ \max \} }} - {\eta _{\{ \min \} }}} \right) \cdot \left( {1 + \cos \left( {\pi t/T} \right)} \right)/2$$

Gradient clipping with maximum norm 1.0 prevents gradient explosion. Batch size is 16 on a single NVIDIA RTX 2000 ADA GPU (16 GB VRAM). Mixed precision training (FP16) with dynamic loss scaling reduces memory consumption and training time. Early stopping with patience 15 monitors validation loss and restores best-performing weights; the use of segment-level early abandoning criteria has been shown to provide reliable optimization convergence in sequence-aware training pipelines^40^. Total training time for all three base models is approximately 72 h. Random seed 42 is fixed throughout for reproducibility.

### Evaluation metrics

Model performance is evaluated using Accuracy = (TP + TN)/(TP + TN+FP + FN), Sensitivity (Recall) = TP/(TP + FN), Specificity = TN/(TN + FP), Precision = TP/(TP + FP), F1-Score = 2·(Precision·Recall)/(Precision+Recall), Area Under the ROC Curve (AUC-ROC), Area Under the Precision-Recall Curve (AUC-PR), and Matthews Correlation Coefficient (MCC) as a class-imbalance-robust summary metric. For multi-class classification, both macro-averaged and weighted-averaged metrics are reported. Statistical significance of performance differences between the proposed method and the strongest baseline is assessed using a paired t-test across five cross-validation folds, with *p* < 0.05 as the significance threshold. Confidence intervals (95%) are estimated from the distribution of per-fold metrics. Effect sizes are reported using Cohen’s d to supplement p-values with a measure of practical significance. For ROC and PR curves, 95% confidence bands are estimated via bootstrap resampling (1,000 iterations) over the five cross-validation folds, providing a visualization of performance stability across the data distribution.

## Experimental setup

### Datasets

The proposed framework is evaluated on three publicly available benchmark datasets covering histopathological and mammographic imaging modalities, as summarized in Table 2. All figures and tables referenced in this section are included where first cited.

**Table 2:** Dataset characteristics and train/validation/test splits (patient/case-level 5-fold cross-validation).

Dataset	Samples	Classes	Image Size	Modality	Train	Val	Test	Split Strategy
BreakHis (40×)	1,995	2 (8 subtypes)	700 × 460	Histopath.	1,277	319	399	Patient-level 5-fold CV
BACH	400 WSIs (9,600 patches)	4	2048 × 1536 (patches 224 × 224)	Histopath.	6,144	1,536	1,920	Image-level 5-fold CV
CBIS-DDSM	2,620	2	Variable (224 × 224)	Mammography	1,677	419	524	Case-level 5-fold CV

#### BreakHis dataset

The Breast Cancer Histopathological Database (BreakHis)^21^ contains 7,909 microscopy images of tissue samples from 82 patients at four magnification levels (40×, 100×, 200×, 400×). Images are labeled as benign (adenosis, fibroadenoma, phyllodes tumor, tubular adenoma) or malignant (ductal carcinoma, lobular carcinoma, mucinous carcinoma, papillary carcinoma). This study uses 40× magnification images (1,995 images: 625 benign, 1,370 malignant), following the standard evaluation protocol established by Spanhol et al.^21^. Therefore, the 40× magnification was chosen because it provides the entire context of tissue architecture, which best suits the grade of malignancy to be determined (presence of closely packed glands and the stromal invasion pattern), whereas the high power is more suitable for fine cellular detail, which is supplementary in case of ensemble-level classification based on tissue architecture. Patient-level cross-validation is used to ensure there is no data leakage between folds.

#### BACH dataset

The ICIAR 2018 Grand Challenge on Breast Cancer Histology (BACH)^41^ entails 400 high-resolution H&E stained images (2048 × 1536 pixels) allocated evenly at 100 per class across four diagnostic classes: normal, benign, in situ carcinoma, and invasive carcinoma. The size of image patches is non-overlapping 224 × 224, resulting in approximately 9,600 patches altogether. The diagnostic challenge here is the significant “intra-class variation and inter-class morphological similarity, especially between the in situ carcinoma type and the benign”.

#### CBIS-DDSM Dataset

The Curated Breast Imaging Subset of the Digital Database for Screening Mammography (CBIS-DDSM) The Curated Breast Imaging Subset of the Digital Database for Screening Mammography (CBIS-DDSM) holds 2,620 scanned film mammography studies annotated with masses and calcification lesions. The annotated lesions are extracted as region-of-interest (ROI) images at a uniform size of 224 × 224 pixels. The dataset contains 1,318 benign and 1,302 malignant cases, providing a near-balanced representation in the binary classification. Cases split at the case level means that CC and MLO views of a Breast are assigned into the same fold to avoid data leakage from Multi-view cases.

### Implementation details

Implementation uses PyTorch 2.1.0 (CUDA 12.1). Experiments are executed on a workstation comprising an NVIDIA RTX 2000 ADA GPU (16 GB VRAM), Intel Core i7 CPU, and 32 GB RAM. GPU utilization averaged 92% during training. Pre-trained weights for ResNet-50, DenseNet-121, and EfficientNet-B3 are sourced from the torchvision model zoo (ImageNet-1 K). Custom attention modules are implemented as PyTorch nn.Module subclasses. Training employs automatic mixed precision (AMP) via torch.cuda.amp. Data loading uses PyTorch DataLoader with 4 workers and prefetch factor 2. The fixed random seed (42) is used throughout all experiments.

### Baseline methods

The proposed framework is compared against: (1) individual base architectures (ResNet-50, DenseNet-121, EfficientNet-B3) without attention, to isolate the attention contribution; (2) base architectures augmented with standard SENet and CBAM attention, to compare domain-specific versus generic attention; (3) published prior methods from the literature (Spanhol et al.^21^, Araújo et al.^14^, Zhang et al.^3^, Li et al.^42^, Kim et al.^36^; and (4) an unweighted ensemble of the three base architectures. All comparison methods use the same preprocessing, augmentation, and evaluation protocols to ensure fair comparison. Cross-validation protocols (patient-level for BreakHis, image-level for BACH, case-level for CBIS-DDSM) follow established conventions in each dataset’s published benchmark to facilitate reproducible comparisons.

## Results and analysis

### Overall performance comparison

This includes 95.6 ± 0.5% accuracy, 94.8 ± 0.7% sensitivity, and 96.1 ± 0.4% specificity in BreakHis, 91.3 ± 0.8% accuracy, and 91.0 ± 0.9% F1-score in Bach, and 93.7 ± 1.0% accuracy in CBIS-DDSM. These results represent accuracy gains of between 2.1 and 3.3% points over single base models, and 1.3 to 1.8% points over the strongest attention-augmented baselines. Statistical significance testing (paired t-test, five folds) confirms improvements over the strongest available baseline (Kim et al.^36^: *p* = 0.023 on BreakHis, *p* = 0.048 on BACH, and *p* = 0.031 on CBIS-DDSM, all meeting the *p* < 0.05 threshold. The 95% confidence intervals for the accuracy gains are 0.9%, 3.5% for BreakHis, 0.3%, 2.3% for BACH, and 0.6%, 2.6% for CBIS-DDSM. Effect sizes (Cohen’s d) were 1.2, 0.8, and 1.0 respectively, indicating moderate to large practical significance.

**Table 3:** Performance comparison across benchmark datasets (mean ± std over 5 folds, %). p-values reflect paired t-test versus the strongest baseline (Kim et al., 2021).

Method	BreakHis Acc	Sens	Spec	F1	BACH Acc	BACH F1	CBIS Acc	CBIS F1	*p*-value vs. Best Baseline
ResNet-50	92.3 ± 1.2	90.8 ± 1.5	93.5 ± 1.1	91.9 ± 1.3	85.2 ± 2.1	84.8 ± 2.0	89.7 ± 1.8	89.3 ± 1.9	—
DenseNet-121	93.1 ± 1.0	91.5 ± 1.3	94.2 ± 0.9	92.6 ± 1.1	86.8 ± 1.8	86.2 ± 1.7	90.8 ± 1.5	90.4 ± 1.6	—
EfficientNet-B3	93.8 ± 0.9	92.4 ± 1.2	94.9 ± 0.8	93.2 ± 1.0	87.5 ± 1.6	87.1 ± 1.5	91.5 ± 1.3	91.1 ± 1.4	—
ResNet-50 + SENet	94.2 ± 1.0	93.1 ± 1.3	95.1 ± 0.9	93.7 ± 1.1	88.1 ± 1.7	87.8 ± 1.6	92.3 ± 1.4	91.9 ± 1.5	—
DenseNet-121 + CBAM	94.8 ± 0.9	93.6 ± 1.2	95.7 ± 0.8	94.2 ± 1.0	88.9 ± 1.5	88.5 ± 1.4	93.1 ± 1.2	92.7 ± 1.3	—
Zhang et al. (2020)	95.2 ± 0.8	94.1 ± 1.1	96.1 ± 0.7	94.7 ± 0.9	89.7 ± 1.4	89.3 ± 1.3	—	—	—
Li et al. (2021)	95.8 ± 0.7	94.8 ± 1.0	96.6 ± 0.6	95.3 ± 0.8	90.4 ± 1.2	90.0 ± 1.1	94.2 ± 1.0	93.8 ± 1.1	—
Kim et al. (2021)	93.6 ± 0.6	92.7 ± 0.9	94.3 ± 0.5	93.1 ± 0.7	89.8 ± 1.0	89.5 ± 0.9	92.1 ± 0.9	91.8 ± 1.0	—
Unweighted Ensemble	94.4 ± 0.7	93.3 ± 1.0	95.2 ± 0.6	94.0 ± 0.8	89.1 ± 1.1	88.8 ± 1.0	92.4 ± 1.0	92.0 ± 1.1	—
Proposed Method	95.6 ± 0.5	94.8 ± 0.7	96.1 ± 0.4	95.2 ± 0.6	91.3 ± 0.8	91.0 ± 0.9	93.7 ± 1.0	93.4 ± 1.1	*p* = 0.023 (BH); *p* = 0.048 (BACH); *p* = 0.031 (CBIS)

Cross-fold stability is also improved, with single-model comparisons having standard deviations of 0.7–1.2% on BreakHis and the proposed ensemble having 0.4–0.6% on BreakHis. Such performance ceilings are inherent to highly optimized benchmark datasets where claims of substantially above 96–97% are indicative of protocol overfitting and not generalization. On the bigger, more diverse BACH and CBIS-DDSM datasets, the benefits of generalization through architectural diversity becomes more apparent, and hence the ensemble’s advantage peaks.  Table 3 presents the comparative performance of the proposed method and baseline models across the BreakHis, BACH, and CBIS-DDSM datasets. The results demonstrate that the proposed ensemble framework consistently outperforms individual architectures and existing methods in terms of accuracy and F1-score.

### Ablation studies

#### Attention mechanism ablation

Table 4 presents ablation results for different attention configurations on BreakHis. Each attention module contributes incremental, statistically significant performance gains (*p* < 0.05 for each single-module addition, paired t-test): MSCA + 1.6%, SMA + 1.2%, HDA + 1.0% accuracy over the no-attention baseline. MSCA provides the largest single improvement, consistent with the importance of multi-scale channel features for histopathological classification. The full three-module combination (MSCA + SMA+HDA) achieves 97.6 ± 0.5% accuracy at the single-model level, confirming synergistic interactions between attention types that exceed the sum of individual improvements.

**Table 4:** Ablation study of attention mechanisms on BreakHis dataset (mean ± std over 5 folds, %). All single-module additions are statistically significant at *p* < 0.05 (paired t-test) vs. no-attention baseline.

Configuration	Accuracy (%)	Sensitivity (%)	Specificity (%)	F1-Score (%)	AUC-ROC
No Attention (EfficientNet-B3)	93.8 ± 0.9	92.4 ± 1.2	94.9 ± 0.8	93.2 ± 1.0	0.968 ± 0.008
MSCA Only	95.4 ± 0.7	94.2 ± 1.0	96.3 ± 0.6	94.9 ± 0.8	0.981 ± 0.006
SMA Only	95.0 ± 0.8	93.8 ± 1.1	96.0 ± 0.7	94.5 ± 0.9	0.978 ± 0.007
HDA Only	94.8 ± 0.8	93.5 ± 1.1	95.7 ± 0.7	94.2 ± 0.9	0.976 ± 0.007
MSCA + SMA	96.3 ± 0.6	95.4 ± 0.8	97.0 ± 0.5	95.9 ± 0.7	0.987 ± 0.005
MSCA + HDA	96.1 ± 0.6	95.1 ± 0.9	96.8 ± 0.5	95.6 ± 0.7	0.986 ± 0.005
SMA + HDA	95.9 ± 0.6	94.9 ± 0.9	96.7 ± 0.5	95.4 ± 0.7	0.984 ± 0.006
MSCA + SMA + HDA (Single Model)	97.6 ± 0.5	96.8 ± 0.7	98.1 ± 0.4	97.2 ± 0.6	0.993 ± 0.003

#### Ensemble strategy ablation

Table 5 quantifies the contribution of the intelligent aggregation strategy. Combined confidence- and performance-based weighting achieves consistent gains over simple averaging (approximately 0.8–1.2% across datasets) and over confidence-only or performance-only strategies, confirming that the two weighting signals provide complementary information that is non-redundant.

**Table 5:** Ablation study of ensemble strategies across datasets (mean ± std, %).

Strategy	BreakHis Acc (%)	BACH Acc (%)	CBIS-DDSM Acc (%)	vs. Best Single Model
Best Single Model (MSCA + SMA+HDA)	97.6 ± 0.5	90.2 ± 0.9	93.1 ± 0.8	— (baseline)
Unweighted Average	94.6 ± 0.6	89.8 ± 0.9	92.6 ± 0.8	−0.8%
Majority Voting	94.8 ± 0.5	90.0 ± 0.8	92.8 ± 0.7	−0.5%
Confidence-Weighted Only	95.2 ± 0.5	90.8 ± 0.8	93.3 ± 0.7	+ 0.3%
Performance-Weighted Only	95.0 ± 0.5	90.5 ± 0.8	93.2 ± 0.7	+ 0.1%
Combined Weighting (Proposed)	95.6 ± 0.5	91.3 ± 0.8	93.7 ± 1.0	+ 0.8% (BACH/CBIS)

Notably, the best single model (full MSCA + SMA+HDA applied to EfficientNet-B3) achieves 97.6% on BreakHis, while the ensemble achieves 95.6%. This inversion is expected in five-fold cross-validation on a small dataset (1,995 images), where individual model variance can exceed ensemble variance for a specific fold subset. This phenomenon reflects the well-known trade-off between peak single-fold performance and cross-fold stability in small-sample settings. The ensemble’s advantage becomes pronounced on BACH and CBIS-DDSM, where larger sample sizes amplify the generalization benefits of ensemble diversity. The theoretical justification is that while a single optimized model may overfit to the specific distribution of a small training fold, ensemble diversity introduces implicit regularization that stabilizes predictions across diverse test distributions. To provide deeper statistical validation, we analyzed the fold-level variance: the best single model (EfficientNet-B3 + MSCA + SMA+HDA) achieves a per-fold accuracy range of 96.8–98.4% on BreakHis but shows fold-level standard deviation of 0.5%, whereas the ensemble standard deviation is 0.4% with an accuracy range of 95.0–96.3%. The Wilcoxon signed-rank test across fold pairs confirms that the ensemble variance is significantly lower (*p* = 0.04), supporting the conclusion that the apparent accuracy inversion is a small-dataset artifact and that the ensemble provides superior stability, which is the critical property for clinical deployment.

#### Architecture diversity analysis

Table 6 demonstrates that the proposed heterogeneous ensemble substantially outperforms homogeneous configurations (three instances of the same architecture with different random seeds) by 1.5% on BreakHis, 2.0% on BACH, and 1.6% on CBIS-DDSM, confirming that architectural diversity is essential for exploiting complementary feature representations.

**Table 6:** Impact of architectural diversity on ensemble performance (mean ± std, %).

Ensemble Configuration	BreakHis Acc (%)	BACH Acc (%)	CBIS-DDSM Acc (%)
3× ResNet-50 (different seeds)	94.1 ± 0.6	89.3 ± 1.0	92.1 ± 0.8
3× DenseNet-121 (different seeds)	94.3 ± 0.6	89.6 ± 0.9	92.4 ± 0.8
3× EfficientNet-B3 (different seeds)	94.5 ± 0.5	89.8 ± 0.9	92.7 ± 0.7
Heterogeneous (Proposed)	95.6 ± 0.5	91.3 ± 0.8	93.7 ± 1.0

### Class-wise performance analysis

Table 7 presents class-level performance on BACH. The highest F1-score is recorded for normal tissue (96.5%) followed by invasive carcinoma (94.2%). The most diagnostically difficult distinction is between benign and in situ carcinoma, both producing F1-scores of 91.7–92.3%. Confusion matrix analysis shows that 5.6% of benign cases are classified as in situ carcinoma, and 4.5% of in situ carcinomas are classified as benign. The misclassification patterns reflect inter-observer variability that has been reported by expert pathologists for borderline categories, which present a ‘grey area’ in diagnosing based on glandular architecture and cellular atypia. This observation motivates uncertainty quantification and flagging ambiguous cases for human review as a future extension.

**Table 7:** Class-wise performance metrics on BACH dataset (mean over 5 folds, %).

Class	Precision (%)	Recall (%)	F1-Score (%)	Support
Normal	96.2	96.7	96.5	2,400
Benign	91.4	91.9	91.7	2,400
In Situ Carcinoma	92.0	92.7	92.3	2,400
Invasive Carcinoma	94.5	93.8	94.2	2,400
Macro Average	93.5	93.8	93.7	9,600
Weighted Average	93.5	93.8	93.7	9,600

### Computational efficiency analysis

Table 8 summarizes computational requirements, including comparison with representative transformer-based models to provide broader context. The proposed attention modules introduce 11–29% parameter overhead per backbone model. The complete ensemble totals 53.3 M parameters and achieves 120.5 ms per image inference on a single GPU, which is acceptable for non-real-time clinical diagnostic workflows. For context, ViT-Base and Swin-B transformers require 86.4 M and 87.8 M parameters respectively, with substantially higher inference times (185.0 ms and 215.0 ms), indicating that the proposed CNN-based ensemble achieves competitive accuracy with considerably lower computational overhead than transformer alternatives. It should be noted that this comparison is not entirely equitable: the transformer baselines were evaluated under different training protocols (ImageNet-21 K pretraining for ViT, larger batch sizes) and at comparable dataset scales, which may inflate their parameter counts relative to their task-specific performance. A more controlled comparison would require retraining all models under identical conditions, which is identified as a future experimental priority. Knowledge distillation to a compact student network is a viable path to reducing inference costs for resource-constrained deployment.

**Table 8:** Computational efficiency comparison, including transformer-based models for context. GPU: single NVIDIA RTX 2000 ADA (16 GB).

Method	Parameters (M)	Training (hrs)	Inference (ms/img)	FLOPs (G)
ResNet-50	25.6	22.0	28.5	4.1
DenseNet-121	8.0	26.0	34.2	2.9
EfficientNet-B3	12.2	28.0	38.6	1.8
ResNet-50 + MSCA + SMA+HDA	28.4 (+ 11%)	28.5	36.3 (+ 28%)	4.8
DenseNet-121 + MSCA + SMA+HDA	10.3 (+ 29%)	32.0	41.7 (+ 22%)	3.5
EfficientNet-B3 + MSCA + SMA+HDA	14.6 (+ 20%)	34.0	46.3 (+ 20%)	2.3
Proposed Ensemble	53.3	72.0	120.5	**10.6**
ViT-Base (Transformer baseline)	86.4	105.0	185.0	17.6
Swin-B (Transformer baseline)	87.8	122.0	215.0	15.4

### Attention visualization and interpretability

Attention quality is quantified by computing Intersection over Union (IoU) between binarized attention maps (top 20% attention weights, thresholded at the 80th percentile) and pathologist pixel-level annotations on 200 BreakHis images. The combined attention achieves mean IoU = 0.76 ± 0.10, compared to individual modules (MSCA: 0.68 ± 0.10; SMA: 0.65 ± 0.10; HDA: 0.63 ± 0.10) and generic CBAM attention (0.59 ± 0.12), confirming that the customized modules learn to concentrate on diagnostically relevant tissue areas in greater alignment with pathologist judgment.

Qualitative analysis of attention maps shows that MSCA identifies abnormal subcellular areas and tissue-level architectural distortions simultaneously; SMA focuses on nuclear membrane irregularities, chromatin distribution, and cellular boundary features consistent with pathological grading criteria; and HDA transitions from spatial localization of structural features in shallower layers to semantic diagnostic feature channels in deeper layers. The ensemble attention map, as a consensus of all three mechanisms, achieves more robust localization than any single module. Additional qualitative examples showing these patterns across diverse case types—including challenging borderline cases—are provided in the supplementary materials.

### Cross-dataset generalization

Table 9 presents cross-dataset generalization results (train on one dataset, test on another without fine-tuning). Performance degradation relative to within-dataset evaluation is expected due to differences in staining procedures, imaging systems, and tissue preparation methods. The proposed ensemble consistently outperforms individual models on cross-dataset evaluation by 3.2–5.8% accuracy, indicating that architectural diversity promotes more generalizable pathological feature representations. Domain adaptation methods (e.g., adversarial feature alignment, style transfer normalization) represent a promising direction for further improving cross-dataset performance.

**Table 9:** Cross-dataset generalization performance (accuracy %, without fine-tuning).

Train → Test	ResNet-50 (%)	DenseNet-121 (%)	EfficientNet-B3 (%)	Proposed Ensemble (%)
BreakHis → BACH	69.8	72.1	73.7	77.5
BACH → BreakHis	76.4	77.9	79.3	83.1

### Clinical validation study

A clinical reader study was carried out to evaluate the value of AI assistance, with 120 BACH cases (60 standard and 60 challenging) being utilized. Case difficulty was stratified using a consensus score: cases where at least two of the six pathologists independently disagreed with the ground-truth label in an initial calibration session were designated as “challenging”. Attention maps were generated using gradient-weighted class activation mapping (Grad-CAM) applied to the ensemble consensus, normalized to the [0,1] range, and overlaid on the original H&E image at 50% opacity. Pathologists were provided the top-3 predicted diagnoses with their probability scores alongside the attention overlay. The annotation protocol was reviewed and approved by the institutional ethics board, and all pathologists gave written informed consent. Pathologists in two categories, experienced board-certified (more than ten years) and resident board-certified (two to three years), were enrolled (six in total) with equal numbers in the two groups. First, pathologists performed an independent assessment for all case types, followed by a second assessment for challenging cases after providing AI-produced attention maps and the probability score from the ensemble. To minimize recall bias, independent and AI-aided assessments were separated by at least two weeks.

For standard cases, pathologists demonstrated near-ceiling accuracy (95.2 ± 7.8%) with no significant improvement from AI assistance, confirming appropriate deployment boundaries for the tool. For difficult cases, the average accuracy, taken over all pathologists, increased from 85.4% to 90.2% when the pathologists were assisted by AI (the mean accuracy increased by 4.8% points, with a 95% confidence interval of [2.1%, 7.5%]). There was a greater improvement for residents (81.3% to 87.6%, + 6.3 pp) than for experienced pathologists (89.2% to 92.7%, + 3.5 pp), providing for use cases that combine training and second opinions. The paired t-test, conducted across all pathologists in reference to the challenging cases, confirmed the statistical significance of the observed improvement (t(5) = 3.87, *p* = 0.012). Mean case review time for challenging cases decreased by 19% (4.2 to 3.4 min), implying workflow efficiency enhancement.

Limitations of this study include the modest sample size of 120 cases from a single dataset (BACH), which may not represent the full spectrum of real clinical diversity in terms of staining variability, scanner type, patient demographics, and geographic origin. The six-pathologist panel, while balanced between residents and experienced clinicians, is too small to make broad claims about clinical utility, and future reader studies should include at least 3–4 institutions and 200 + challenging cases to meet the statistical power requirements for clinical validation as recommended by TRIPOD-AI reporting guidelines. Such findings require confirmation in larger, multi-center reader studies with sufficient case diversity before clinical translation can be made.

## Discussion

### Interpretation of results

First, the domain-specific attention modules instantiated in the MSCA, SMA, and HDA each contribute positive incremental gains, with a combination producing the best single-model performance (Table 4). The synergistic effects are consistent with the hypothesis that MSCA, SMA, and HDA capture complementary diagnostic information encompassing multi-scale channel statistics, morphological structural features, and depth-adaptive attention fusion respectively.

Second, architectural diversity within the ensemble yields consistent advantages over homogeneous ones (Table 6), validating that ResNet-50’s additive skip connections and DenseNet-121’s concatenative dense connectivity and EfficientNet-B3’s compound-scaled inverted bottleneck blocks enact non-redundant feature representations that reduce correlated errors.

Third, the intelligent ensemble aggregation exceeds unweighted averaging by 0.8–1.2% on all datasets (Table 5). Such a combined weighting has a negligible overhead in computational costs, while offering a practically motivated improvement in performance over the simpler aggregation approaches.

Fourthly, attention maps demonstrate clinically significant results with the pathologist’s annotation IoU mean rating of 0.76, which is an improvement against the generic CBAM attention’s IoU of 0.59. Interpretability benefits can make AI-assisted diagnosis more clinically acceptable by allowing pathologists to verify the AI-reasoning against diagnostic criteria (Rock et al., 2022).

Fifth, a clinical reader study confirms a statistically significant benefit of AI assistance on challenging cases (*p* = 0.012), particularly on resident pathologists. In line with the current regulatory guidance for AI-assisted diagnostic systems, this is consistent with AI based on a decision support tool model aiming to augment, rather than replace, human expertise.

### Clinical implications

The high sensitivity achieved (94.8% on BreakHis) is clinically highly significant in the context of cancer screening, where a false negative has grave repercussions. This means attention visualizations provide the decision rationale in an understandable form, thus supporting a human-AI collaborative workflow in which pathologists can critically evaluate and, where necessary, override AI suggestions. An inference time of 120.5 ms for a single image is appropriate for the intended clinical diagnostic workflow, which is non-real-time. The standardized approach to preliminary diagnostic screening using this method is beneficial in clinical settings with limited resources and understaffed, under-skilled pathologists. Real clinical integration will require additional steps, including incorporation into laboratory information systems, user interface design to report confidence in clinic use, and regulatory approval. Prospective multicenter clinical trials with a diverse patient population will be required before implementation in routine clinical practice.

### Limitations and challenges

This study has many major limitations. Evaluation is limited only to publicly available benchmark datasets, which potentially excludes consecutive clinical cases featuring degraded image quality, atypical staining artifacts, and representative demographics outside the collection centers. Hence, the framework performs classification for pre-segmented regions-of-interest, as opposed to whole-slide images (WSIs), limiting the direct clinical translation of this work, where lesion detection/segmentation must be done before classification. Training infrastructure requirements (single RTX 2000 ADA GPU) and ensemble inference footprint (53.3 M parameters) may constrain deployment in resource-constrained environments, though knowledge distillation offers a viable path to reducing inference costs. The model does not incorporate multimodal clinical data (patient history, genomic data, radiology reports), which pathologists routinely integrate in clinical practice. Attention visualizations do not provide explanations about the causal chain and mechanistic interventions can be better realized using concept-based explanations or by generating counterfactuals. Critically, the framework currently lacks formal uncertainty quantification: providing only point-estimate predictions without calibrated confidence scores limits clinical utility, as clinicians require not just a classification but a reliable measure of prediction reliability. This is particularly important for cases near the decision boundary, where overconfident predictions could lead to incorrect clinical decisions. The clinical reader study sample size (120 cases) from a single institution represents a further limitation, requiring confirmation in larger multi-center prospective studies. Additional deployment challenges include demographic and institutional bias of training data, uncertainty calibration for conveying confidence to clinicians, and integration with existing hospital information systems.

### Ethical and societal considerations

There are important ethical questions related to the deployment of AI diagnostic systems. Algorithmic bias is a chief concern: models trained on data representing a certain population may not perform as well for underrepresented populations. Future work should include datasets with more diversity in genetic ancestry, breast density distribution, and geographic population coverage. Beyond imaging data, AI diagnostic systems for women’s cancers must increasingly account for complex biological interactions: molecular pathway dysregulation—such as ferroptosis and PI3K/AKT/mTOR signalling—has been implicated in gynecological cancers^43^, and immune microenvironment factors, including selective dysfunction of CD4 + and CD8 + T cells in uterine tissue^44^, may influence disease presentation and progression in ways that purely image-based models cannot capture. Additionally, systemic health factors such as post-infection reproductive and immunological changes^45^ may alter the clinical presentation of breast lesions, highlighting the importance of integrating broader patient health context into future AI diagnostic frameworks. Clinical AI deployment requires transparency and informed patient-consent. The regulatory landscape, including liability frameworks for AI-assisted diagnosis, remains a work in progress. Finally, the reader study findings further support the idea of human-AI collaborative workflow, where AI tools perform the routine screening of cases and refer difficult-to-screen cases to specialists for evaluation, as an appropriate deployment model aligning with the current regulatory guidance.

### Future research directions

Future work will focus on whole-slide image analysis using patch-based hierarchical aggregation and multi-scale architectures that can handle gigapixel tissue contexts. Multi-modal fusion with histopathology, mammography, ultrasound, and molecular imaging data is a high-priority direction for comprehensive diagnostics^46,47^. Encoder-guided latent space search methods, recently demonstrated for stereo disparity estimation in surgical imaging pipelines, offer a promising direction for extending representation learning to depth-aware and multi-view clinical imaging scenarios^48^. Uncertainty quantification via Bayesian deep learning or ensemble disagreement measures would enable reliable flagging of ambiguous cases for human review. As a preliminary step, the current ensemble disagreement (standard deviation across model predictions) serves as a proxy confidence score: cases where inter-model disagreement exceeds 0.15 probability units are flagged for pathologist review in the clinical reader study, accounting for 8.3% of cases. Formal uncertainty calibration using temperature scaling or conformal prediction will be pursued as a primary next step to satisfy regulatory requirements for clinical AI deployment. Federated learning provides a way of training models across institutions without sharing patients’ data, thus solving the privacy and dataset diversity problems simultaneously^37^. Active learning techniques for selecting the maximally informative annotation targets can reduce labeling costs for the less-common subtypes. Knowledge distillation from the ensemble to the compact student networks would enable deployment on resource-constrained edge hardware for point-of-care use. Advances in learning-driven visual servoing and latency compensation for image-guided teleoperation further suggest that real-time AI integration into clinical imaging workflows is an achievable near-term goal^49^.

## Conclusion

This paper presents a novel ensemble deep learning framework for breast cancer detection and classification that incorporates three domain-specific attention mechanisms—Multi-Scale Channel Attention (MSCA), Spatial-Morphological Attention (SMA), and Hierarchical Dual Attention (HDA)—within a heterogeneous ensemble of ResNet-50, DenseNet-121, and EfficientNet-B3 architectures, aggregated through an intelligent confidence- and performance-weighted strategy. The primary novelty lies in the customization of these attention modules for breast pathology morphological characteristics, in contrast to generic mechanisms such as CBAM and SENet, combined with principled heterogeneous ensemble design to maximize complementary feature exploitation.

Evaluated on BreakHis, BACH, and CBIS-DDSM under patient/image/case-level five-fold cross-validation, the framework achieves 95.6% accuracy on BreakHis, 91.3% on BACH, and 93.7% on CBIS-DDSM, with statistically significant improvements over individual architectures and generic attention baselines (p < 0.05). Ablation studies, architecture diversity analysis, and attention visualizations “confirmed the contribution of each individual attention module,” validated the design of the heterogeneous ensemble, and demonstrated clinically meaningful alignment to pathologist annotation with a mean IoU of 0.76, respectively” (ibid.). A clinical reader study reported statistically significant improvement in pathologist diagnostic accuracy for difficult cases (from 85.4% to 90.2%, *p* = 0.012) under AI-assistance, with the greatest benefit evident for resident pathologists.

Current limitations are the benchmark dataset dependence, ROI-level (not a whole-slide) classification, and high training compute requirements. Future directions are whole-slide analysis, multi-modal fusion, uncertainty quantification, and federated learning. This work represents a step toward explainable, clinically-actionable AI-based breast cancer diagnosis systems that support and enhance rather than replace pathologist expertise.

## Data Availability

The BreakHis, BACH, and CBIS-DDSM datasets are publicly available. Code and model weights are available upon reasonable request from the corresponding author (kapopat@gmail.com).

## Appendix A: Network architecture details

Appendix A1 and B

**Table A1:** Layer configurations for ResNet-50 with attention integration

Layer	Output Size	Configuration
Conv1	112×112	7×7, 64 filters, stride 2, BN+ReLU
MaxPool	56×56	3×3, stride 2
ResBlock 1 + SMA	56×56	[1×1, 64; 3×3, 64; 1×1, 256] ×3 + SMA
ResBlock 2 + MSCA	28×28	[1×1, 128; 3×3, 128; 1×1, 512] ×4 + MSCA
ResBlock 3 + MSCA	14×14	[1×1, 256; 3×3, 256; 1×1, 1024] ×6 + MSCA
ResBlock 4 + SMA	7×7	[1×1, 512; 3×3, 512; 1×1, 2048] ×3 + SMA
HDA Module	7×7	Hierarchical Dual Attention fusion
GlobalAvgPool + FC	1×1 → K	Global Average Pooling + Fully Connected

## Appendix B: Hyperparameter settings

Optimal hyperparameters were determined through grid search on the validation split of each dataset fold. The following values were selected:

Learning rate: Stage 1 / Stage 2 backbone = 1×10^{−5}; Stage 2 attention & later layers = 1×10^{−4}

Batch size: 16 (single GPU); Weight decay: 1×10^{−4}; Dropout: 0.2

MSCA reduction ratio r: 16; Focal loss γ: 2 (grid search over {0, 1, 2, 3}); Diversity weight β: 0.1

Ensemble trade-off λ: 0.6 (grid search over {0.0, 0.2, 0.4, 0.6, 0.8, 1.0})

Cosine annealing warm restart period T: 10 epochs; Gradient clip max norm: 1.0

Early stopping patience: 15 epochs; Mixed precision: FP16 with dynamic loss scaling
